# Nodule-Enriched GRETCHEN HAGEN 3 Enzymes Have Distinct Substrate Specificities and Are Important for Proper Soybean Nodule Development

**DOI:** 10.3390/ijms18122547

**Published:** 2017-11-28

**Authors:** Suresh Damodaran, Corey S. Westfall, Brian A. Kisely, Joseph M. Jez, Senthil Subramanian

**Affiliations:** 1 Department of Agronomy, Horticulture and Plant Science, South Dakota State University, Brookings, SD 57007, USA; suresh.damodaran@sdstate.edu (S.D.); brian.kisely@ndsu.edu (B.A.K.); 2 Department of Biology, Washington University in St. Louis, St. Louis, MO 63130, USA; cswestfa@wustl.edu; 3 Department of Biology and Microbiology, South Dakota State University, Brookings, SD 57007, USA

**Keywords:** *Gretchen Hagen 3* (*GH3*), auxin, indole-3-acetic acid (IAA), soybean (*Glycine max*), nodule, artificial microRNA

## Abstract

Legume root nodules develop as a result of a symbiotic relationship between the plant and nitrogen-fixing rhizobia bacteria in soil. Auxin activity is detected in different cell types at different stages of nodule development; as well as an enhanced sensitivity to auxin inhibits, which could affect nodule development. While some transport and signaling mechanisms that achieve precise spatiotemporal auxin output are known, the role of auxin metabolism during nodule development is unclear. Using a soybean root lateral organ transcriptome data set, we identified distinct nodule enrichment of three genes encoding auxin-deactivating GRETCHEN HAGEN 3 (GH3) indole-3-acetic acid (IAA) amido transferase enzymes: *GmGH3-11/12*, *GmGH3-14* and *GmGH3-15*. In vitro enzymatic assays showed that each of these GH3 proteins preferred IAA and aspartate as acyl and amino acid substrates, respectively. *GmGH3-15* showed a broad substrate preference, especially with different forms of auxin. Promoter:GUS expression analysis indicated that *GmGH3-14* acts primarily in the root epidermis and the nodule primordium where as *GmGH3-15* might act in the vasculature. Silencing the expression of these *GH3* genes in soybean composite plants led to altered nodule numbers, maturity, and size. Our results indicate that these GH3s are needed for proper nodule maturation in soybean, but the precise mechanism by which they regulate nodule development remains to be explained.

## 1. Introduction

Spatiotemporal auxin output is a combination of tightly regulated biosynthesis, catabolism, inactivation, activation, transport, and signaling [1,2]. The major form of auxin in plants, indole-3-acetic acid (IAA), is primarily synthesized via the two-step Indole pyruvic acid (IPA) pathway [3]. In this pathway, tryptophan is converted to IPA by TRYPTOPHAN AMINO TRANSFERASE OF ARABIDOPSIS (TAA) and IPA is metabolized to IAA by YUCCA flavin monoxygenases [4,5]. It was recently revealed that 2-oxoindole-3-acetic acid (oxIAA) is the major catabolite of IAA in Arabidopsis and rice [6,7]. A dioxygenase enzyme that catabolizes IAA to oxIAA has also been identified [6,8,9,10]. Different biologically inactive forms of IAA including amide-linked peptide conjugates, amide-linked amino acid conjugates, and ester-linked sugar (carbohydrate) conjugates have been identified in plant tissues [11,12,13]. Conjugation of different amino acids leads to different downstream fates for IAA. For example, IAA-alanine and IAA-leucine conjugates can be hydrolyzed to release free IAA in specific cell types for proper embryo development in Arabidopsis [14,15]. IAA conjugates of aspartate (IAA-Asp) and glutamate appear to be catabolic forms that typically cannot be hydrolyzed back to IAA [2]. The fate of conjugated forms of IAA varies from species to species [2]. Therefore, conjugation of IAA is a key regulatory step that dictates the levels of free (active) IAA pools and thus spatiotemporal auxin output during plant development.

Members of the GRETCHEN HAGEN3 (GH3) family of acyl amido transferase enzymes can conjugate IAA to amino acids [2]. The first *GH3* gene was identified in soybean through a screen for auxin responsive gene expression [16]. Subsequently, *GH3* family members were identified in other plant species including Arabidopsis, and found to play critical roles in plant development through the conjugation of various plant hormones [2]. For example, a change in local auxin pool is achieved at the site of organ development or in response to biotic/abiotic interaction through conjugation of IAA by GH3 proteins [17,18,19,20]. A gain of function mutation in an Arabidopsis *GH3* gene, *wes1-D* conferred resistance against multiple factors and a loss of function mutation in the same gene led to reduced resistance [19]. A gain-of-function mutation in another Arabidopsis *GH3* gene, *ydk1-D* led to reduced root length and lateral root density because of altered auxin activity [21]. An activation-tagged Arabidopsis line with increased expression of GH3.9 exhibited increased sensitivity to IAA, resulting in reduced root growth [22]. Recently, the X-ray crystal structures of IAA- and jasmonate-conjugating GH3 proteins were determined. This has revealed key features of substrate recognition and to the re-classification of the GH3 enzyme family into different groups based on the preference of the acyl acid substrate [23,24]. Group II GH3 proteins catalyze IAA-amino acid conjugation and alter the free IAA pool to regulate various plant developmental programs in Arabidopsis and other plant species [25].

Symbiotic nodule development in legumes such as soybean is also influenced by auxin. Nodule development results from a symbiotic relationship between the plant and nitrogen-fixing rhizobia bacteria. Rhizobia colonize plant root hairs, and after initial signal exchange to ensure host-symbiont compatibility, plant developmental pathways are activated to enable nodule organogenesis in the root cortex. Auxin signaling has been implicated in both root hair as well as cortical responses during nodule development [26,27,28,29,30,31]; however, distinct mechanisms might contribute to overall auxin output in these cell types. The distribution and levels of auxin in the root cortex may be distinct in different legumes (reviewed by [32]). There are two major classes of legume nodules (reviewed by [33,34]). Indeterminate nodules characterized by the presence of a persistent meristem with an oblong mature nodule are produced by *Medicago truncatula* (barrelclover), *Pisum sativum* (peas), and *Trifolium repens* (white clover). Determinate nodules that lack a persistent meristem with a spherical mature nodule are produced by *Lotus japonicus*, *Glycine max* (soybean), and *Phaseolus vulgaris* (common bean). Altered auxin signaling is reported to affect root hair responses to rhizobium inoculation in soybean and *M. truncatula* [26,27]. A local auxin maximum occurs in the root cortex at the site of initiation of both determinate and indeterminate nodules. Evidence for this comes primarily from auxin-responsive marker gene expression, and at least one study where auxin levels were measured at the site of nodule initiation [35,36,37,38]. The type of mechanism involved and the degree of auxin accumulation or output required appear to differ between these two types of nodules [32]. Inhibition of rootward auxin transport at the site of nodule initiation by flavonoids is crucial for indeterminate nodule formation [17,39]. Expression patterns of genes encoding PIN auxin efflux transporters, and phenotypes of *PIN*-RNAi plants in *M. truncatula* also indicate a key role for the auxin transport machinery during indeterminate nodule development [40,41]. On the other hand, inhibition of auxin transport does not appear to be crucial for determinate nodule formation [36,42]. While an auxin maximum appears to be crucial for nodule initiation, enhanced sensitivity to auxin inhibits both determinate and indeterminate nodule formation [26,28,29,30,31].

Determinate and indeterminate nodules also display similarities and differences in the overall distribution of auxin activity during nodule development. As mentioned above, local auxin activity indicated by marker gene expression occurs in the nodule initials and nodule primordia of determinate nodules (soybean and *L. japonicus*), as well as indeterminate nodules (white clover and *M. truncatula*) [28,35,37,39]. Auxin responsive gene expression is significantly diminished/absent in the infection zone of determinate nodules; however, the nodule meristem and invasion zone of indeterminate nodules continue to display auxin response gene expression. In mature nodules, auxin activity is detectable in the vasculatures of both determinate and indeterminate nodules (e.g., [28,35]). Therefore, precise regulation of auxin activity appears to occur during nodule development. While auxin transport appears to dictate auxin distribution during initiation of indeterminate nodules, it is unclear what mechanisms contribute to it during determinate nodule initiation. Multiple microRNA-regulated AUXIN RESPONSE FACTORs (ARFs) that might act in concert to dictate precise spatiotemporal auxin sensitivity during nodule development are also known [26,28,29,30,31]. However, the role of auxin metabolism in regulating auxin homeostasis during nodule development remains unclear. Flavonoids that accumulate at the sites of nodule initiation can inhibit peroxidases capable of degrading auxin and this has been suggested as a possible mechanism for auxin accumulation in these tissues [43]. Transient induction of *TRYPTOPHAN AMINOTRANSFERASE RELATED1*, a paralog of *TAA*, occurs in response to rhizobium inoculation in *L. japonicus* [38]. In soybean, we have shown enrichment of *YUCCA*, *GH3*, and IAA oxidase gene expression in emerging nodules (Damodaran et al. unpublished data [44]). In *M. truncatula*, the expression of several *GH3* genes is induced in *Sinorhizobium meliloti* treated roots [45]. Similarly, rhizobium-responsive expression of auxin conjugate hydrolases capable of hydrolyzing the ester bonds of IAA-glucose and thus releasing free IAA have also been reported [46]. Rhizobia are also capable of synthesizing auxin [47,48]. Therefore, expression of auxin-modifying enzymes during nodule development is likely to enable the plant to efficiently regulate rhizobia-derived auxin as well. While these observations suggested that local auxin metabolism might contribute to auxin output during nodule initiation and development, no functional evidence existed for this hypothesis. We sought to identify the roles of auxin-conjugating GH3 proteins in soybean nodule development.

Here, we identified three *GH3* genes with preferential expression during nodule development and characterized their enzymatic activity through in vitro assays. We also evaluated their expression patterns in roots and nodules of soybean, and their functional significance during nodule development by knocking down their expression using artificial microRNAs. We show that these GH3 proteins have distinct expression patterns in soybean, and show highest activity towards IAA-Asp conjugation, but have distinct specificities especially for other acyl substrates. Suppression of GH3 protein activity led to alterations in nodule number and nodule size indicating that these enzymes play important roles in soybean nodule development likely via their effect on auxin homeostasis.

## 2. Results

### 2.1. Identification of Nodule-Enriched GmGH3 Genes

**Figure 1:**
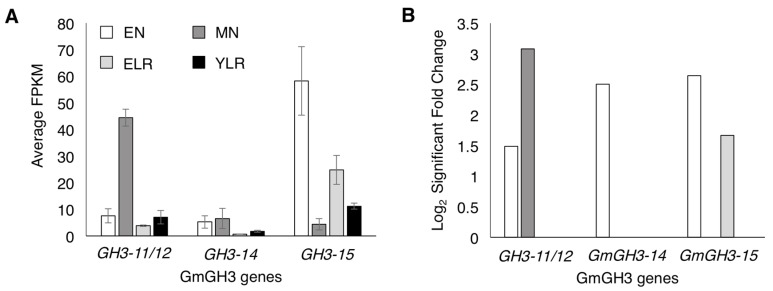
Expression of *GmGH3* genes in soybean root lateral organs. (**A**) Expression levels of GmGH3s in emerging nodule (EN), mature nodule (MN), emerging lateral root (ELR), and young lateral root (YLR) tissues. Data shown are average expression values from three biological replicates obtained using RNA-seq. Normalized gene expression levels based on RNA-seq read counts are shown in FPKM (fragments per kilobase of transcript per million mapped reads). Error bars indicate SD; (**B**) Enrichment of *GmGH3* gene expression in the same four tissue types relative to adjacent root tissues. Data shown are statistically significant log_2_ fold change values vs. the respective control root segments from three biological replicates. See Table S1 for additional details.

Nodule-enriched *GH3* genes in soybean were identified from our RNA-seq dataset on emerging nodules (EN), mature nodules (MN), emerging lateral roots (ELR), and young lateral roots (YLR) (Damodaran et al. unpublished data [44]; Table S1). Adjacent root segments above and below these organs were used as age- and rhizobium inoculation-appropriate controls to determine *GH3* genes specifically enriched in nodules versus lateral roots at two different stages of development (Table S1). Among a total of five *GH3* genes that showed enrichment in nodule tissues, three with high expression and enrichment in either emerging or mature nodule tissues were selected (highlighted in Table S1; Figure 1A). These three showed the highest expression values with two of the three GH3s showing nodule-specific enrichment. The three genes were named as *GmGH3-11/12* (Glyma11g05510 (a1. v1.1), Glyma.11g051600 (a2. v1.1)), *GmGH3-14* (Glyma01g39780 (a1. v1.1), Glyma.01g190600 (a2. v1.1)), and *GmGH3-15* (Glyma12g17510 (a1. v1.1), Glyma.12g141000 (a2. v1.1)) based on the nomenclature/classification of the 25 soybean *GH3* genes previously [23]. Gene IDs in parenthesis correspond to those of soybean genome assembly release a1.v1.1 and a2.v1.1 (www.phytozome.net). The three *GmGH3* genes used in this study were classified under group II GH3s that catalyze IAA conjugation.

*GmGH3-11/12* was expressed in all four lateral organ tissues examined with highest expression in mature nodule tissues (Figure 1A). It showed enrichment only in nodule tissues with a 3.1-fold log_2_ fold change in MN followed by 1.5 in EN (Figure 1B). Expression of *GmGH3-14* and *GmGH3-15* was detected in all four lateral organ tissues. Despite near equal expression in EN and MN tissues, *GmGH3-14* expression was enriched only in EN tissues (Figure 1A,B). *GmGH3-15* was expressed at relatively higher levels than *GmGH3-14* in general, and was enriched in both EN and ELR with log_2_ fold change values of 2.6 and 1.7, respectively (Figure 1A,B).

### 2.2. Nodule-Enriched GmGH3s Show Distinct Acyl Substrate Specificities

**Figure 2:**
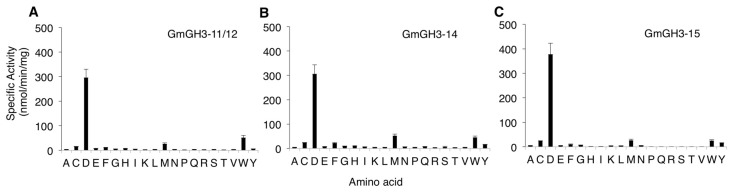
Amino acid substrate preference of nodule enriched GmGH3s. Specific activities of (**A**) GmGH3-11/12; (**B**) GmGH3-14; and (**C**) GmGH3-15 with IAA as acyl substrate with each of the 20 different amino acids denoted by single letter IUPAC codes. Data shown are the average specific activities from three replicate assays and error bars indicate SD.

Enzymatic activities of the nodule enriched GH3 proteins were evaluated using in vitro enzyme kinetics assays. Full-length proteins were expressed in bacterial cells, and purified for biochemical assays in which the conjugation of the 20 amino acids to IAA were evaluated. GmGH3-11/12, GmGH3-14, and GmGH3-15 all displayed a clear preference for conjugation of IAA to aspartate (Figure 2). *GmGH3-11/12* had a specific activity of 296.2 nmol·min^−1^·mg·protein^−1^ with aspartate and much lower specific activities with tryptophan (51.12 nmol·min^−1^·mg·protein^−1^) and methionine (26.05 nmol·min^−1^·mg·protein^−1^) (Figure 2A). GmGH3-14 had a specific activity of 305.9 nmol·min^−1^·mg·protein^−1^ with aspartate, and much lower rates with methionine (51.8 nmol·min^−1^ mg protein^−1^) and tryptophan (44.4 nmol·min^−1^·mg·protein^−1^) (Figure 2B). The specific activity profile of GmGH3-15 was similar with conjugation of IAA to aspartate (377.8 nmol·min^−1^·mg·protein^−1^) as the primary function, although methionine (26.1 nmol·min^−1^·mg·protein^−1^), cysteine (24.4 nmol·min^−1^·mg·protein^−1^), and tryptophan (24.9 nmol·min^−1^·mg·protein^−1^) were accepted as amino acid substrates (Figure 2C).

**Figure 3:**
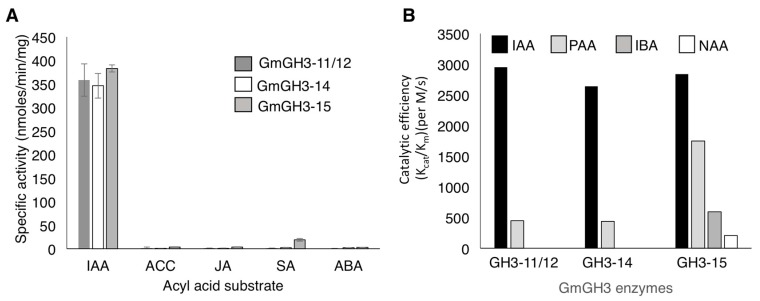
Acyl substrate preference of nodule-enriched GmGH3s. (**A**) Specific activity of GmGH3-11/12, GmGH3-14, and GmGH3-15 with aspartate as aminoacid substrate, and the plant hormones indole-3-acetic acid (IAA), aminocyclopropane carboxylic acid (ACC), jasmonic acid (JA), salicylic acid (SA), and abscisic acid (ABA) as acyl substrate. Data shown are averages of three replicate assays and error bars indicate SD; (**B**) Catalytic efficiency of GmGH3-11/12, GmGH3-14, and GmGH3-15 with aspartate as amino acid substrate, and different forms of auxin: IAA, phenyl acetic acid (PAA), indole butyric acid (IBA), and naphthalene acetic acid (NAA) as acyl substrate. See Table S2 for additional details.

GH3 proteins are capable of generating conjugates of different plant hormones including jasmonic acid, IAA and other auxins, and benzoate-derived compounds [23]. Therefore, steady-state kinetic assays were performed using IAA, the ethylene precursor 1-aminocyclopropane carboxylic acid (ACC), abscisic acid (ABA), jasmonic acid (JA), and salicylic acid (SA) to further examine substrate preference (Figure 3A). GmGH3-11/12, GmGH3-14, and GmGH3-15 exhibited little to no activity with ACC, ABA, JA, and SA (Figure 3A).

Although IAA is the primary auxin in many plants, several different forms of auxin are present in plant tissues and the levels of auxin analogs vary between species and between different tissues [12,49]. To determine the substrate preference of the three GmGH3 proteins with different auxins, kinetic assays were performed using most abundant natural forms of auxin, IAA, phenyl acetic acid (PAA), and indole butyric acid (IBA), and the synthetic auxin, naphthalene acetic acid (NAA). As mentioned above, all three GmGH3s showed high catalytic efficiency towards IAA (Figure 3B; Table S2). The catalytic efficiencies (k_cat_/K_m_) of GmGH3-11/12, GmGH3-14, and GmGH3-15 with IAA were 2950 M^−1^·s^−1^, 2640 M^−1^·s^−1^, and 2840 M^−1^·s^−1^, respectively. Each of the soybean GH3 proteins were also capable of using PAA as substrate, although not as efficiently as IAA. Of the three proteins, GmGH3-15 displayed a 3-fold higher k_cat_/K_m_ for PAA compared to the other two enzymes (Figure 3B; Table S2). GmGH3-15 also used IBA (k_cat_/K_m_ = 592 M^−1^·s^−1^) and NAA (k_cat_/K_m_ = 207 M^−1^·s^−1^) as substrates, whereas the other two GH3s did not show any activity with these auxins (Figure 3B; Table S2). These results suggest that all these GH3 proteins likely conjugate IAA with aspartate to mark IAA for degradation in soybean. While GmGH3-11/12 and GmGH3-14 had comparable substrate preferences, GmGH3-15 showed a broader auxin substrate preference. 

### 2.3. Distinct Spatio-Temporal Expression Patterns of GmGH3-14 and GmGH3-15 in Soybean Roots and Nodules

We characterized in detail the expression patterns and functional roles of *GmGH3-14* and *GmGH3-15* genes in soybean roots and nodules. Technical difficulties in cloning the promoter region precluded the characterization of *GmGH3-11/12* expression patterns. The promoter region upstream (~1900 bp) of the coding sequences of both *GmGH3-14* and *GmGH3-15* were fused to bacterial uidA gene encoding a β-glucuronidase (GUS) and the transcriptional fusions were expressed in soybean hairy root composite plants. The expression patterns of GmGH3-14p:GUS and GmGH3-15p:GUS were monitored at 0, 10, and 14 days post rhizobium inoculation (dpi) through histochemical staining for GUS activity.

**Figure 4:**
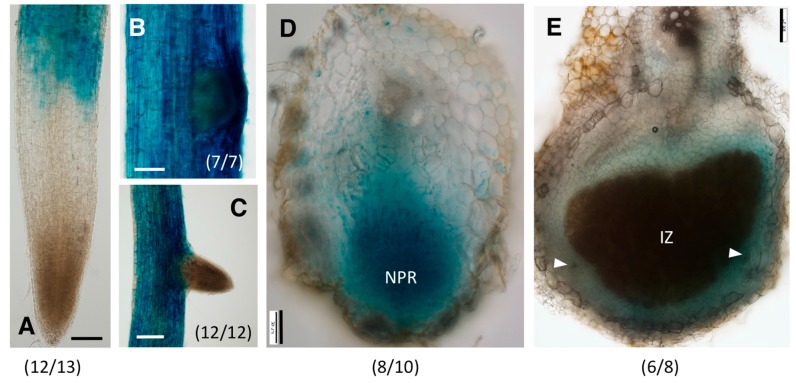
Expression patterns of GmGH3-14p:GUS in soybean roots and nodules. (**A**–**C**) Expression of GmGH3-14p:GUS in (**A**) root tips; (**B**) mature root region with a lateral root primordium; and (**C**) mature root region with a young lateral root; (**D**,**E**) Expression of GmGH3-14p:GUS in (**D**) emerging nodule (transverse section at 10 dpi); and (**E**) mature nodule (transverse section at 17 dpi). NPR-nodule primordium; IZ–infection zone; Arrowheads indicate nodule vascular bundles. The number of independent transgenic roots/nodules showing the representative staining pattern out of the number of roots/nodules examined is indicated in each panel. Scale bars: (**A**,**B**,**E**) 100 μm; (**C**) 200 μm; (**D**) 50 μm.

At 0 dpi, GmGH3-14p:GUS was expressed primarily in the root epidermis above the meristematic region (Figure 4A). There was no detectable gene expression in the root tip, including the root cap, quiescent center, and the root meristem, until the differentiation zone. In mature regions of the root, the expression of the construct was primarily in the root epidermis and was more prominent in the lateral root primordia (Figure 4B). As the lateral root emerges, the expression of GmGH3-14p:GUS is not detectable in the ELR at the root tip similar to that of the primary root tips (Figure 4C). The epidermal expression of GmGH3-14p:GUS made it difficult to clearly image the early cortical cell division during nodule development, but in emerging nodules, GUS expression was observed in the nodule primordia (Figure 4D). As the nodule matures the expression of GmGH3-14p:GUS was localized to the nodule parenchyma including the nodule vasculature (Figure 4E).

**Figure 5:**
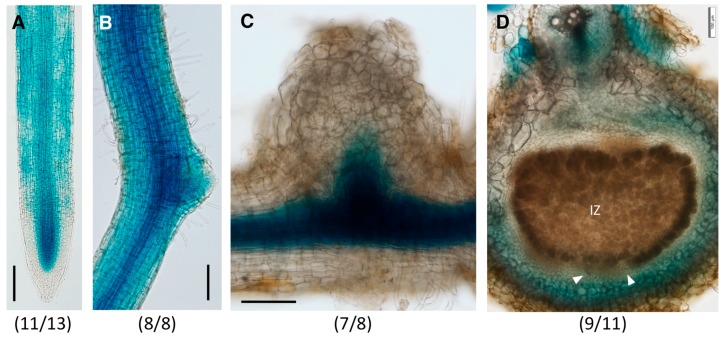
Expression patterns of GmGH3-15p:GUS in soybean roots and nodules. (**A**,**B**) Expression of GmGH3-14p:GUS in (**A**) root tips; and (**B**) mature root region with an emerging lateral root; (**C**,**D**) Expression of GmGH3-15p:GUS in (**C**) emerging nodule at 10 dpi and (**E**) mature nodule (transverse section at 17 dpi). IZ–infection zone; Arrowheads indicate nodule vascular bundles. The number of independent transgenic roots/nodules showing the representative staining pattern out of the number of roots/nodules examined is indicated in each panel. Scale bars: 100 μm.

*GmGH3-15* was expressed in the root meristematic region, specifically above the quiescent center cells and in the elongating cells of the root vasculature (Figure 5A). GUS staining was absent in the root cap, as well as young epidermal and cortex cells of the root meristem. In the mature regions of the root, GmGH3-15p:GUS expression was detectable in the root epidermis and was prominent in the vasculature (Figure 5B). Similar to *GmGH3-14*, the promoter of *GmGH3-15* was also active in the lateral root primordia (Figure 5B). In emerging nodules, GmGH3-15p:GUS expression was observed at the junction of root and nodule where initiation of nodule vasculature development occurs (Figure 5C). There was no detectable expression in the nodule primordium or other nodule tissues. As the nodule matured, the expression was primarily localized in the parenchyma region and tissues surrounding the sclerid layer (Figure 5D). Expression was largely absent in parenchyma cells closest to the infection zone, unlike that of GmGH3-14p:GUS, which was expressed throughout the parenchyma. Overall, *GmGH3-14* and *GmGH3-15* have distinct spatiotemporal expression patterns in root tips and emerging nodules. Both genes were generally expressed in the nodule parenchyma of mature nodules with subtle differences.

### 2.4. GmGH3-14 and GmGH3-15 Are Important for Proper Nodule Numbers in Soybean

**Figure 6:**
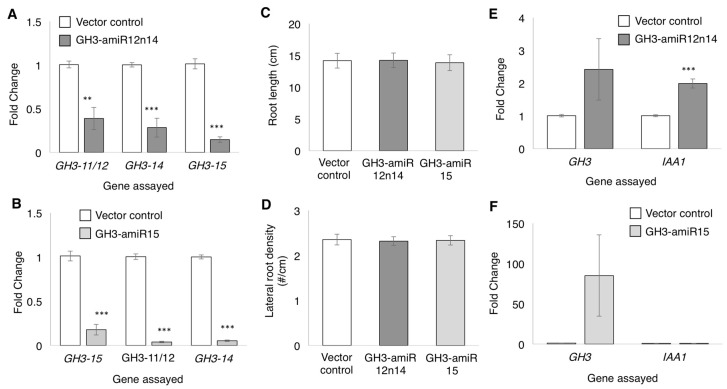
Suppression of *GmGH3* expression by artificial microRNAs. (**A**,**B**) Expression of target GmGH3 genes in roots expressing (**A**) GH3-amiR12n14 and (**B**) GH3-amiR15 relative to vector control roots; (**C**) Root length and (**D**) lateral root density of vector control roots and roots expressing GH3-amiRs. Data shown are averages (*n* = 21) and error bars indicate SEM. No significant difference observed using Student’s *t*-test; (**E**,**F**) Expression of auxin response marker genes GH3 and IAA1 in roots expressing (**E**) GH3-amiR12n14; and (**F**) GH3-amiR15, relative to vector control roots. Expression levels shown in (**A**,**B**,**E**,**F**) were assayed by RT-qPCR and normalized to that of Actin in each sample. Data shown are average relative expression values (fold change vs. vector control) from three biological replicates and error bars indicate the range of possible value based on SD between replicates. ** *p* < 0.01, *** *p* < 0.001, Wilcoxon-Mann-Whitney test.

To evaluate the role of GmGH3 proteins in soybean nodule development, we sought to knock down their expression in soybean composite plants. High sequence similarity among family members precluded the use of RNAi; therefore, artificial miRNAs to independently silence *GmGH3-14* and *GmGH3-15* were designed ([50]; Figure S1). The high sequence similarity between *GmGH3-14* and *GmGH3-11/12* made it difficult to design a specific artificial miRNA construct against *GmGH3-14*. Therefore, the amiRNA against *GmGH3-14* was expected to silence both *GmGH3-11/12* and *GmGH3-14* and was named GH3-amiR12n14. The amiRNA targeting *GmGH3-15* was named GH3-amiR15. The amiRNA sequences were synthesized using *gma-miR164* pri-miRNA as backbone (Figure S1) and expressed using the constitutive CsVMV promoter [51] in soybean hairy root composite plants. The “empty vector”, pCAMGFP-CsVMV:GW was used to generate vector control hairy root composite plants. To evalute amiRNA-mediated gene silencing, the expression of GmGH3 genes were quantified using RT-qPCR (Figure 6). The expression levels of the corresponding targets were significantly reduced in roots expressing GH3-amiR12n14 and GH3-amiR15 compared to the vector control roots (Figure 6A,B). However, the amiRNAs also led to the reduction in expression levels of non-target *GH3* genes (Figure 6A,B). GH3-amiR12n14 led to reduction in the levels of *GmGH3-15*; and GH3-amiR15 led to a significant reduction in the expression levels of *GmGH3-11/12* and *GmGH3-14* (Figure 6A,B). GH3-amiR15 led to >95% reduction in *GmGH3-11/12* and *GmGH3-14* expression where as GH3-amiR12n14 led to ~60–70% reduction of these genes. Despite the silencing of non-target *GH3* genes, we expected that suppression of *GH3* expression in these roots might lead to a reduction in IAA-Asp formation, resulting in an increased active auxin pool. As a proxy for increased active auxin levels, we measured root length and lateral root density (number of lateral roots/cm of primary root) in these roots. There was no significant differences in these phenotypes in either of the GH3-amiR expressing roots relative to the vector control roots (Figure 6C,D). We also assayed the expression of auxin response marker *GH3* (not targeted by the amiRNA) and *INDOLE ACETIC ACID1* (*IAA1*) as a proxy for increased auxin levels. We observed 2.4-fold and 72-fold increases in expression of auxin-responsive *GH3* in GH3-amiR12n14 roots and GH3-amiR15 roots, respectively. However, The differences were not statistically significant due to high variation between biological replicates (Figure 6E,F). *IAA1* showed a statistically significant 2-fold higher expression in GH3-amiR12n14 roots, but no change in GH3-amiR15 roots compared to vector control roots. While physiological assays such as root length and lateral root density are likely to indicate cumulative effects of potential changes in auxin levels, gene expression markers are typically indicative of responses at the time of tissue harvest. This is likely the reason for inconsistency between markers, and large variation among replicates.

**Figure 7:**
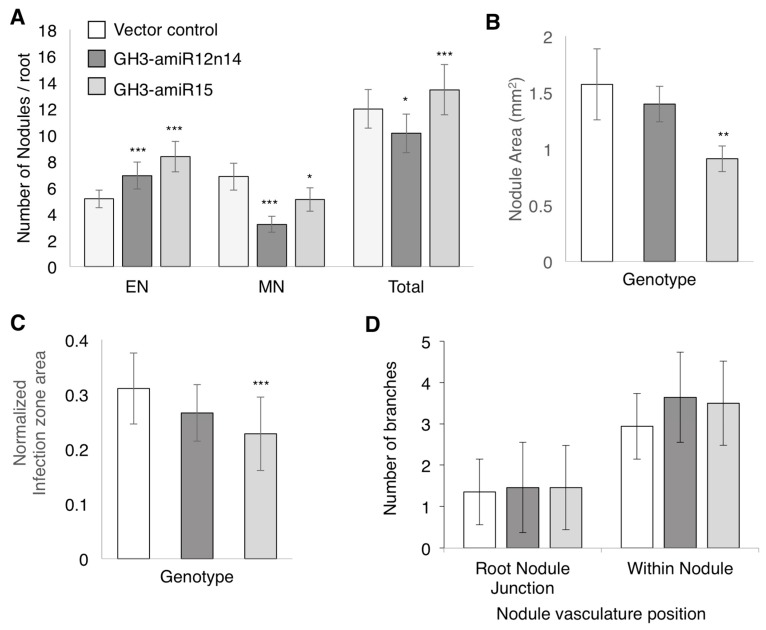
Nodule numbers and morphology in GH3-amiR expressing soybean roots. (**A**) Numbers of emerging, mature, and total nodules in vector control, GH3-amiR12n14, and GH3-amiR15 expressing roots at 17 dpi. Data shown are the averages of at least 68 roots for each construct from three independent experiments. Error bars indicate SE. * *p* < 0.05, *** *p* < 0.001, Poisson distribution test; (**B**) Nodule area; and (**C**) normalized infection zone size of mature nodules from vector control, GH3-amiR12n14, and GH3-amiR15 expressing roots. Data shown are averages of at least 15 nodules each from three biological replicates. Error bars indicate SE. ** *p* < 0.01, *** *p* < 0.001, Wilcoxon-Mann-Whitney test; (**D**) Number of vasculature branches detectable at the root-nodule junction and within the nodule in transverse sections of mature nodules from vector control, GH3-amiR12n14, and GH3-amiR15 expressing roots. Data shown in C are averages of at least 15 nodules each from three biological replicates. Error bars indicate SE. Student’s *t*-test.

To determine the role of the amiRNA in nodule development, composite plants over-expressing GH3-amiRNAs were inoculated with B. japonicum and the numbers of emerging and mature nodules were counted at 14–17 dpi (Figure 7A). In roots over-expressing GH3-amiR12n14, there was a significant increase in the number of emerging nodules and a significant reduction in the number of mature nodules compared to the vector control. Roots expressing GH3-amiR15 also displayed a significant increase in the number of emerging nodules and a reduction in the number of mature nodules. The effects on the two amiRNAs on total nodule numbers were distinct from each other. While GH3-12n14amiRNA caused a reduction in total nodule numbers, GH3-15amiRNA caused an increase in total nodule number (Figure 7A). This was due to the difference in magnitude of increase in emerging nodules and decrease in mature nodules between the two amiRNAs. GH3-amiR12n14 caused a relatively lower magnitude of increase in emerging nodule numbers, but a much higher reduction in mature nodule numbers versus GH3-amiR15. This data suggested that the expression of GmGH3 genes during nodule development is crucial for proper nodule organogenesis and maturation.

### 2.5. GmGH3s Influence Nodule Size in Soybean

To evaluate the effect of suppressing *GmGH3* genes on nodule morphology, median cross sections of mature nodules perpendicular to the root were imaged, and nodule and infection zone area were measured using ImageJ (Figure 7B–D and Figure S2). In roots over-expressing GH3-amiR12n14, there was no significant change in either the nodule area or the infection zone area compared to the nodules from vector control roots (Figure 7B). In roots overexpressing GH3-amiR15, there was a significant reduction in both the nodule and infection zone area (Figure 7C). The nodule sections were also stained with phloroglucinol and evaluated for nodule vasculature development by counting the number of visible vasculature branches at the nodule-root junction and in the nodule parenchyma (Figure 7D and Figure S2). Typically 1–2 vascular strands are visible at the nodule-root junction, and 3–5 strands are visible in the parenchyma indicating branching of the vasculature in nodule tissues. There was no significant difference in the number of vasculature branches at either position in GH3-amiR12n14 or GH3-amiR15 over-expressing roots. Overall, our results suggest that GmGH3 proteins regulate nodule number, infection zone size, and nodule size.

## 3. Discussion

Auxin appears to play both positive and negative roles during nodule development depending on the level of auxin output, developmental stage, and type of legume nodule. Auxin perception by TIR/AFB family of F-box proteins appears to be crucial for root hair curling during determinate nodule development in soybean [27]. On the other hand, in *M. truncatula* (that produced indeterminate nodules) *arf16-1* mutants and lines over-expressing miR390, both of which had enhanced sensitivity to auxin had impaired root hair responses [26,31]. Enhanced response to auxin due to suppression of repressor auxin response factor transcription factors (ARF10/16/17) inhibits nodule development in soybean, although root hair responses and nodule initial cell division were unaffected [28]. Similar conclusions on the relationship between auxin sensitivity and nodule formation were suggested by other studies in soybean (ARF8, [30]) and *M. truncatula* (ARF3/4, [31]). In particular, suppression of repressor ARF transcription factors in the nodule primordium tissues using an *ENOD40:miR160* construct inhibited nodule formation suggesting that enhanced auxin response in the primordium might inhibit formation of additional nodules in soybean [28]. We observed increased numbers of emerging nodules and reduced numbers of mature nodules in soybean composite plants over-expressing GH3amiR constructs. This was unexpected, as one would have expected reduced nodulation resulting from an increase in free auxin levels due to reduced IAA-Asp conjugation in these roots. While we did observe an overall reduction in nodulation in GH3-amiR12n14 plants, we observed an increased number of total nodules in GH3-amiR15 plants.

Two issues made it difficult for us to clearly interpret these results: non-specific silencing of GH3s by the amiRNAs, and broad-substrate specificity of GmGH3-15. Despite bioinformatics predictions and careful design, both amiRNAs significantly reduced the expression of all three GH3 proteins. GH3-amiR15 plants had a >95% reduction in expression levels of *GmGH3-11/12* and *GmGH3-14* where as it was ~60–70% in GH3-amiR12n14 plants; however, the level of suppression of *GmGH3-15* was comparable between GH3-amiR12n14 and GH3-amiR15 plants. Therefore, the phenotypic difference between GH3-amiR12n14 and GH3-amiR15 plants is likely to have resulted from difference in suppression of *GmGH3-11/12* and *GmGH3-14*. Promoter:GUS assays showed that *GmGH3-14* is highly expressed in the root epidermis, and soybean gene expression atlas showed that the expression of both *GmGH3-11/12* and *GmGH3-14* are induced in root hairs upon rhizobium inoculation (Table S1). Reduced expression of these genes is likely to have resulted in an increase in free auxin levels in root hairs upon rhizobium inoculation. We speculate that this would have resulted in increased infection and nodule formation because increased auxin response appears to promote rhizobial infection at least in soybean [27]. *GmGH3-14* is also expressed in the nodule primordium, and its suppression in these cells should have led to more free auxin and suppression of nodule development. The apparent contradiction might have resulted from suppression of more than one GH3 with distinct expression patterns by the amiRNA constructs. For example, the construct also silenced *GmGH3-12* which is highly expressed and enriched in mature nodules. Nevertheless, we observed a reduction in number of mature nodules in both GH3-amiR expressing roots. In GH3-amiR15 roots where the expression levels of all three *GH3* genes were strongly reduced, we also observed reduction in nodule size. Together these data indicate that the GmGH3s evaluated in this study play a key role in nodule maturation and contribute to nodule size. It was also interesting to note that *GmGH3-14* and *GmGH3-15* were expressed in vascular tissues where typically high auxin activity is observed. It is possible that these genes act to establish threshold auxin levels for vascular differentiation. Generation of specific knock-outs in each GH3 through CRISPR/Cas-mediated gene editing might offer a more clear answer to the role of each of these *GH3* genes in nodule development.

Secondly, GmGH3-15 displayed a broad substrate specificity and much higher catalytic efficiency than other characterized GH3s. Since GmGH3-15 showed substantial activity towards PAA, and IBA, it is possible that the activity of more than one auxin and even other hormones might have been affected in the GH3-amiR roots (see below). The ability of GmGH3-15 to utilize different forms of auxin such as IAA, PAA, IBA, and NAA was reminiscent of the broad substrate specificity of the Arabidopsis GH3.5 (AtGH3.5) protein [52]. Indeed, phylogenetic analyses indicate that both *GmGH3-15* and *AtGH3.5* belong to the same orthoclade [53]. GmGH3-15 had a much higher catalytic efficiency on IAA (Figure 3B) compared to AtGH3.5 [52]. Similarly, while AtGH3.5 had near equal catalytic efficiencies between IAA and PAA, GmGH3-15 was about 3-fold more efficient with IAA over PAA. The abundance of PAA in plants is near equal or even higher than that of IAA, although the former is relatively less active than IAA [54]. In Arabidopsis, over-expression of *AtGH3.5* or gain of function mutations resulted in reduced free IAA and PAA levels and increased IAA-Asp and PAA-Asp levels [19,52,55], but the relative ratio of PAA-Asp vs. PAA was much higher than that of IAA-Asp vs. IAA. It was suggested that PAA-Asp might be more stable or a storage form [52]. Therefore, we speculate that silencing of *GmGH3-15* might have resulted in altered PAA accumulation as well in GH3-amiR roots. It is possible that PAA in addition to IAA might play a role in soybean nodule development.

GmGH3-15 also displayed high catalytic efficiency towards benzoic acid (BA), and 4-hydroxy benzoic acid (4-HBA), and low, but detectable activity towards SA (Table S3). Arabidopsis *GH3.5* gain of functions mutants (*wes1-D* and *gh3.5-1D*) accumulate higher levels of SA during pathogen challenge, and over-expression of *AtGH3.5* also led to increase in SA and SA-Asp [19,52,55]. It has been suggested that at least part of this SA might have been derived through conversion of BA or BA-Asp to SA [52]. Therefore, we speculate that GmGH3-15 might regulate SA levels in soybean. SA inhibits nodule development, but its site of action is unclear. Exogenous SA clearly inhibited both rhizobial association with root hairs and nodule primordium formation in indeterminate nodule forming legumes, but not in determinate nodule forming legumes [56]. However, reduction in endogenous SA levels by expressing *nahG* (a bacterial SA hydroxylase gene) increased rhizobial infection as well as nodule formation in both determinate and indeterminate nodule forming legumes. When plants were co-treated with nod factors and SA, root hair deformation responses were unaffected, but primordium initiation was significantly reduced [56,57] suggesting that SA might primarily inhibit cortical cell responses during nodule development. Given that gain of GH3.5 function in Arabidopsis led to increased SA accumulation in Arabidopsis, one might expect reduced SA and BA accumulation in *GmGH3-15*-silenced soybean roots. This is also plausible explanation for increased emerging nodule formation in these roots. While PAA has not been directly implicated in legume nodule development, a balance between positive effect of PAA and negative effect of SA has been suggested during actinorhizal nodule development [58]. It is possible that GmGH3-15 influences nodule development through its action on more than one plant hormone. Precise tissue-specific assays of the target hormones and conjugates are expected to clarify the specific role of GmGH3-15 in soybean nodule development. In conclusion, our results clearly show that these GH3 proteins are important for proper nodulation in soybean while the precise mechanism by which they regulate nodule development remains to be explained.

## 4. Materials and Methods

### 4.1. Protein Expression, Purification, and Enzyme Assays

The coding sequences of *GmGH3-12*, *GmGH3-14*, and *GmGH3-15* were amplified by PCR using high fidelity polymerase enzymes from soybean (*Glycine max* cv. Williams82) root cDNA as template. Amplicons were cloned into a pET-28a bacterial expression vector and verified by sequencing. The coding sequence of *GmGH3-15* had a silent mutation (T101T caused by ACT > ACC) and *GmGH3-11* had a S492P mutation (TCT > CCT) compared to the reference sequence in multiple independent clones suggesting that these were not PCR artifacts. The N-terminally His-tagged fusion proteins of the GH3s were expressed in *Escherichia coli* BL21-CodonPlus-RP cells (Stratagene/Agilent, Santa Clara, CA, USA). The fusion protein was purified following cell lysis by sonication using nickel-based affinity purification, and size-exclusion chromatography, as described for other GH3 proteins [52,59]. The enzymatic activity of the three purified GH3 enzymes were assayed spectrophotometrically as previously described [52,59].

### 4.2. Cloning for Promoter:GUS and Artificial miRNA

The promoter region upstream (~1900 bp) of the coding sequences of *GmGH3-14* and *GmGH3-15* were amplified by PCR using high fidelity polymerase enzymes, cloned into the pCR8-GWTOPOTA vector (Thermofisher Scientific, Waltham, MA, USA), and verified by sequencing. The promoter fragments were cloned in to the destination vector, pCAMGFP-GW:GUS using Gateway LR clonase II enzyme mix following the manufacturer’s protocol (Thermofisher Scientific, Waltham, MA, USA) to obtain pCAMGFP-GmGH3-14p:GUS and pCAMGFP-GmGH3-15p:GUS.

Artificial miRNAs (amiRNAs) were designed by submitting the sequences of target and non-target GH3 genes to the artificial miRNA designer web tool available at (http://wmd3.weigelworld.org/cgi-bin/webapp.cgi) [50]. The top most amiRNA from the resulting output was selected for silencing *GmGH3-15*. Only a common artificial miRNA was available for both *GmGH3-11/12* and *GmGH3-14*. The mature artificial miRNA sequences were inserted in to the pri-miRNA sequence of gma-miR164a using gene synthesis (Figure S1; Table S4). The resulting artificial miRNA precursors were amplified by PCR using high fidelity polymerase enzymes, cloned into pCR8GWTOPOTA vector (Thermofisher Scientific, Waltham, MA, USA), and verified by sequencing. The amiRNA precursors were cloned in to the destination vector, pCAMGFP-CsvMV:GW using Gateway LR clonase II enzyme mix following the manufacturer’s protocol (Thermofisher Scientific, Waltham, MA, USA) to obtain pCAMGFP-CsVMV:GH3-amiRNA vectors. The artificial miRNAs were driven by the constitutively active Cassava vein mosaic virus CVP2 promoter (CsVMV) in these constructs.

The vectors were transformed in to *Agrobacterium rhizogenes* (K599) through electroporation using a Bio-Rad Gene pulser (Bio-Rad Laboratories, Hercules, CA, USA) with settings 25 µF, 400 Ω and 1.8 kV in a 0.1 cm gap cuvette.

### 4.3. Plant Material and Growth Conditions

*Glycine max* cv. Williams-82 seeds were surface sterilized by rinsing with 8% Clorox for 4 min followed by 70% ethanol for 4 min. The seeds were immediately rinsed thoroughly with distilled water 8–12 times to remove any residual bleach and/or ethanol. Seeds were germinated in 4′′ pots filled with 3:1 vermiculite:perlite and watered with Hoagland plant nutrient solution. The plants were grown in a vertical growth chamber with controlled environmental conditions as follows: 16 h light and 8 h dark with a day and night temperature of 25 °C and 20 °C respectively.

### 4.4. Plant Transformation and Nodulation Assay

Hairy root composite plant transformation was performed following the protocol described previously [60] using 12–14 days old soybean seedlings as explants and infecting them with *A. rhizogenes* cells transformed with constructs of interest. Twenty-one days after transformation, the plants produced adventitious roots and *A. rhizogenes*-induced transgenic roots. GFP positive roots carrying the transgene of interest were selected by screening for epifluorescence using the FITC filter in an Olympus SZX16 microscope (Olympus Corporation, Shinjuku, Tokyo, Japan).

For nodulation assays, the screened plants were transferred to 4′′ pots filled with sterilized 3:1 vermiculite: perlite mix. Five days post transfer, the plants were inoculated with *B. japonicum USDA110* cells re-suspended in nitrogen free plant nutrient solution (N^−^ PNS) to OD_600 nm_ of 0.08 [28,61]. About 25 mL of this suspension was added uniformly to each pot. For mock-inoculated plants, the same quantity of N^−^ PNS was applied. Transgenic roots were harvested under an epifluorescence microscope at 14–17 dpi and the nodules were counted. Nodules were classified as “emerging” if they appeared as a bump on root surface and “mature” if they were completely protruded out of the root surface. The statistical significance of difference in nodule numbers if any between amiRNA and vector control roots was determined using zero inflated Poisson distribution package available in R statistical software.

### 4.5. GUS Staining and Microscopy

For evaluation of spatiotemporal promoter:GUS expression, GFP-positive transgenic roots were subjected to GUS histochemical staining at 0, 7, 10 and 14 dpi. Roots were incubated in GUS staining buffer [62] containing the chromogenic substrate X-Gluc (concentration of 0.5 mg·mL^–1^) overnight or until blue staining was visible on the roots, at room temperature. To avoid diffusion of GUS signal, and to arrest the enzymatic reaction the roots were subjected to dehydration with a series of ethanol dilutions from 10% to 70%. Before imaging the GUS-stained roots, they were rehydrated through a series of ethanol in the reverse from 70% to 10% and finally collected in water. For evaluation of GUS expression in nodules, free hand transverse sections of nodules were made using a fresh, sharp razor blade where needed. Whole mounts or sections were mounted on a glass slide in sterile water and covered with a thin cover slip for imaging. The samples were imaged using an Olympus SZX16 microscope under white light trans-illumination or with an Olympus BX-53 upright microscope (Olympus Corporation, Shinjuku, Tokyo, Japan).

### 4.6. Phloroglucinol Staining

To determine nodule morphology, mature nodules from transgenic roots harvested at 14–17 dpi were used. Free hand transverse sections of mature nodules along with the root were stained with a saturated solution of phloroglucinol (Sigma-Aldrich, St. Louis, MO, USA) prepared freshly before staining by dissolving the dye in 20% HCl. The dyes enables visualization of lignified tissues such as vascular bundles which stain bright red in color. The nodule vasculature within the nodule and at the junction of root and nodule was manually counted from these images and the statistical significance of any differences was evaluated using Student’s *t*-test in Microsoft Excel. Measurement of nodule area was performed in *Image J* [63] by manually drawing a border around the nodule area and infection zone using the free hand tool (Figure S2). Statistical significance of any differences was determined using Mann-Whitney-Wilcox test package in R.

### 4.7. Quantitative Gene Expression Analysis

To determine the silencing of target *GH3* gene expression by artificial miRNAs and to measure the expression of auxin response genes, *GH3* and *IAA1*, root tips were collected from un-inoculated roots of vector control and artificial miRNA-expressing roots in triplicate, and frozen in liquid nitrogen. Total RNA was isolated from these tissues, and gene expression was assayed using RT-qPCR as previously described [28,29]. Gene expression levels were normalized to that of house-keeping genes *CONS7*, *CONS15*, *ACTIN*, or *CONS6* independently [64]. Data shown are relative to that of *ACTIN*. Results obtained using other house-keeping genes yielded similar conclusions. The statistical significance of any difference in gene expression was determined using Mann-Whitney-Wilcox test. The sequences of primers used in this study are presented in Table S4.

## Supplementary Materials

Supplementary materials can be found at www.mdpi.com/1422-0067/18/12/2547/s1.

## Abbreviations

GH3	*Gretchen Hagen 3*
IAA	indole-3-acetic acid
IPA	indole pyruvic acid
oxIAA	2-oxoindole-3-acetic acid
ARF	auxin response factor
ACC	1-aminocyclopropane carboxylic acid
ABA	abscisic acid
JA	jasmonic acid
SA	salicylic acid
PAA	phenyl acetic acid
IBA	indole butyric acid
NAA	naphthalene acetic acid
GUS	β-glucuronidase
RT-qPCR	reverse transcription-quantitative polymerase chain reaction
BA	benzoic acid
4-HBA	4-hydorxy benzoic acid
N^−^ PNS	nitrogen-free plant nutrient solution
