# Supplementary material for: Nodule-Enriched GRETCHEN HAGEN 3 Enzymes Have Distinct Substrate Specificities and Are Important for Proper Soybean Nodule Development
Source: Int J Mol Sci. 2017 Nov 28;18(12):2547. doi: 10.3390/ijms18122547 (PMC5751150; doi:10.3390/ijms18122547)
Supplement: Supplementary file 1 [file ijms-18-02547-s001.zip › ijms-239460_Supplementary_Figures.pdf]

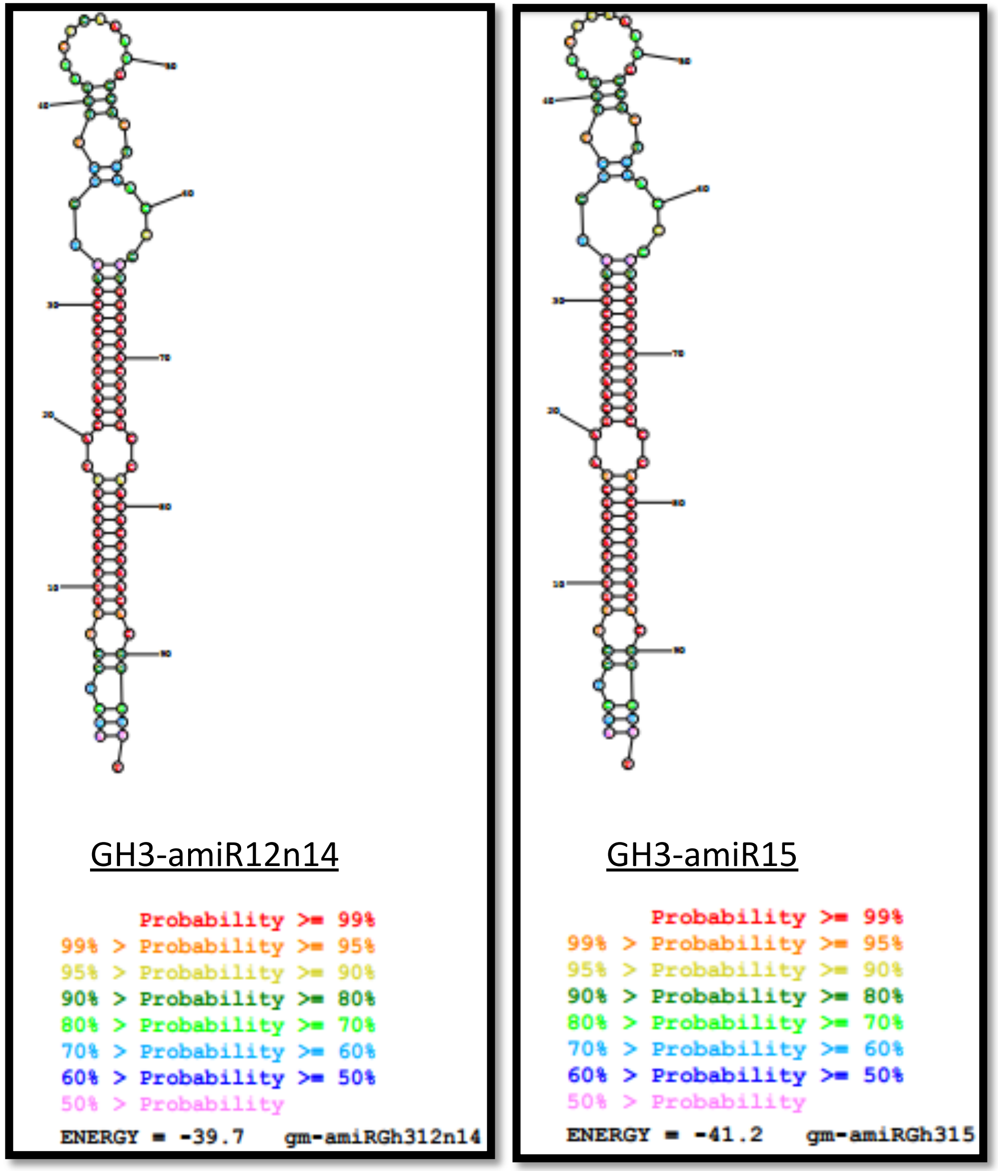

Figure S1. Secondary structures of artificial miRNA precursors (GH3-amiR12n14 and GH3-amiR15). The designed artificial miRNA sequences were inserted in to the backbone of gma-miR164a replacing the original miRNA and miRNA* sequences. Image were generated using https://rna.urmc.rochester.edu/RNAstructureWeb/Servers/Predict1/Predict1.html

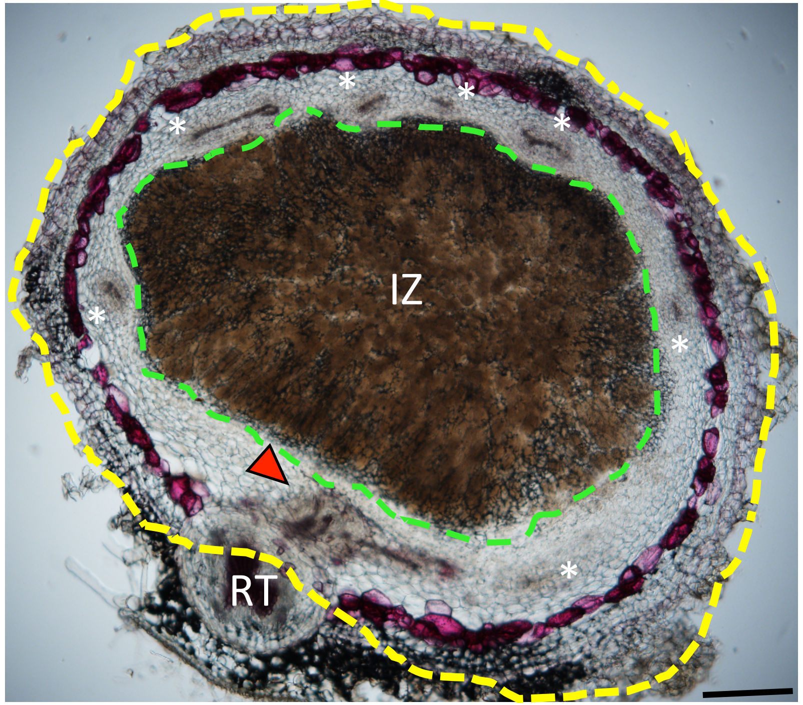

Figure S2. Median transverse section of a mature nodule stained with phloroglucinol. The nodule infection zone boundary is marked with a green dotted line and the nodule boundary is marked with a yellow dotted line. The nodule vascular bundle at the root-nodule junction is indicated using a red arrowhead and the vascular bundles within the nodule are indicated using aterisks. IZ − Infection Zone; RT − root vasculature. Scale bar − 100µm.
